# microRNA-378 promotes mesenchymal stem cell survival and vascularization under hypoxic–ischemic conditions *in vitro*

**DOI:** 10.1186/scrt520

**Published:** 2014-11-23

**Authors:** Yue Xing, Jingying Hou, Tianzhu Guo, Shaoxin Zheng, Changqing Zhou, Hui Huang, Yuyang Chen, Kan Sun, Tingting Zhong, Jingfeng Wang, Honghao Li, Tong Wang

**Affiliations:** The Sun Yat-sen Memorial Hospital of Sun Yat-sen University, 107 Yanjiang Xi Road, Guangzhou, Guangdong 510120 China; Guangdong Province Key Laboratory of Arrhythmia and Electrophysiology, 107 Yanjiang Xi Road, Guangzhou, Guangdong 510120 China; Thyroid and Vascular Surgery, Sun Yat-sen Memorial Hospital of Sun Yat-sen University, 107 Yanjiang Xi Road, Guangzhou, Guangdong 510120 China; Department of Emergency, Sun Yat-sen Memorial Hospital of Sun Yat-sen University, 107 Yanjiang Xi Road, Guangzhou, Guangdong 510120 China

## Abstract

**Introduction:**

Mesenchymal stem cells (MSCs) transplantation has been demonstrated to be an effective strategy for the treatment of cardiovascular disease. However, the low survival rate of MSCs at local diseased tissue reduces the therapeutic efficacy. We therefore investigated the influence of MicroRNA-378 (miR-378) transfection on MSCs survival and vascularization under hypoxic-ischemic condition in vitro.

**Methods:**

MSCs were isolated from bone marrow of Sprague–Dawley rats and cultured *in vitro*. The third passage of MSCs were divided into the miR-378 group and control group. For the miR-378 group, cells were transfected with miR-378 mimic. Both groups experienced exposure to hypoxia (1% O_2_) and serum deprivation for 24 hours, using normoxia (20% O_2_) as a negative control during the process. After 24 hours of reoxygenation (20% O_2_), cell proliferation and apoptosis were evaluated. Expressions of apoptosis and angiogenesis related genes were detected. Both groups were further co-cultured with human umbilical vein endothelial cells to promote vascular differentiation for another 6 hours. Vascular density was assessed thereafter.

**Results:**

Compared with the control group, MSCs transfected with miR-378 showed more rapid growth. Their proliferation rates were much higher at 72 h and 96 h under hypoxic condition (257.33% versus 246.67%, *P* <0.01; 406.84% versus 365.39%, *P* <0.05). Cell apoptosis percentage in the miR-378 group was significantly declined under normoxic and hypoxic condition (0.30 ± 0.10% versus 0.50 ± 0.10%, *P* <0.05; 0.60 ± 0.40% versus 1.70 ± 0.20%, *P* <0.01). The miR-378 group formed a larger number of vascular branches on matrigel. BCL2 level was decreased accompanied with an upregulated expression of BAX in the two experimental groups under the hypoxic environment. BAX expression was reduced in the miR-378 group under the hypoxic environment. In the miR-378 group, there was a decreased expression of tumor necrosis factor-α on protein level and a reduction of TUSC-2 under normoxic environment. Their expressions were both downregulated under hypoxic environment. For the angiogenesis related genes, enhanced expressions of vascular endothelial growth factorα, platelet derived growth factor-β and transforming growth factor-β1 could be detected both in normoxic and hypoxic-ischemic conditions.

**Conclusion:**

MiR-378 transfection could effectively promote MSCs survival and vascularization under hypoxic-ischemic condition in vitro.

## Introduction

Cardiovascular disease with resultant heart failure and malignant arrhythmia is a major cause of morbidity and mortality worldwide [1]. In recent years, stem cell therapy has emerged as a novel strategy for the treatment of cardiovascular disease, and its beneficial efficacies have been confirmed by various preclinical and clinical trials [2–5]. Bone marrow mesenchymal stem cells (MSCs), which have great potential for proliferation and differentiation, have the capacity to differentiate into cardiomyocytes and vascular cells under appropriate conditions [6, 7]. Implantation of MSCs results in regeneration of cardiomyocytes and neovascularization in myocardial infarction. Moreover, these cells can confer protection to ischemic tissues through the release of paracrine factors, thus providing a promising therapeutic modality for repair of the injured heart [8–10]. A series of clinical trials have already shown that MSCs treatment can attenuate ventricular remodeling and improve cardiac function in patients with myocardial infarction and chronic heart failure [4, 5, 11, 12]. However, inferior survival of MSCs under hypoxic condition reduces their therapeutic efficacy [13, 14]. Low survival rates of the donor cells could be due to inflammation, ischemia and apoptosis [15, 16]. Therefore, how to enhance MSCs survival and promote their vascularization in the local hypoxic environment becomes a main issue that needs to be addressed in order to improve the clinical benefits of MSCs transplantation.

microRNAs (miRNAs) are small noncoding RNAs that control gene expression post-transcriptionally. They exert functions over a wide range of cellular processes, including the regulation of stem cell pluripotency and differentiation [17]. Manipulation of miRNAs in stem cells may enhance their capacity for cell survival and vascular regeneration [18, 19]. miRNAs can promote MSCs differentiation into cardiovascular cell lineage and affect MSCs-mediated cardiac repair [20]. microRNA-378 (miR-378) is a specific miRNA that can induce angiogenesis in tumors [21]. Experimental studies show that miR-378 transfection significantly enhances cell viability and inhibits cell apoptosis [22, 23]. In addition, miR-378 is a newly described member of the cardiac-enriched miRNAs modulating cardiac growth during the postnatal period [24]. Its deficiency leads to the development of cardiac hypertrophy [25]. A distinct reduction of miR-378 in patients with heart failure has been reported, implying that it may also participate in the disease progression of heart failure [26].

miR-378 is closely associated with stem cell survival and vascular differentiation. In this study, MSCs were transfected with miR-378 and exposed to normal and hypoxic–ischemic conditions to observe their survival, proliferation and apoptosis. Vascular density was evaluated and the expression of molecules related to apoptosis and vasculogenesis was detected.

## Materials and methods

### Ethics statement

One-month-old Sprague–Dawley rats were obtained from the Animal Experimental Center of the Sun Yat-sen University (Guangzhou, China). All animal handling and procedures were performed in accordance with protocols approved by the Animal Ethics Committee of Sun Yat-sen University (201210016).

### Isolation and culture of bone marrow mesenchymal stem cells

Bone marrow cells were collected from 1-month-old Sprague–Dawley rats by flushing femurs and tibias under sterile conditions. The cells were then cultured (37°C, 5% carbon dioxide) in 25 cm^2^ culture flasks with complete culture medium supplemented with 10% fetal bovine serum penicillin (100 IU/ml) and streptomycin (100 μg/ml). On the third day of culture, the medium was replaced and nonadherent cells were removed. Adherent cells gaining 90% confluence were passaged using 0.25% trypsin–ethylenediamine tetraacetic acid (Mediatech, Hendon, VA, USA) and then maintained in complete medium. Characteristics of MSCs were identified by fluorescence-activated cell sorting as reported previously [27].

### microRNA-378 transfection

miR-378 mimic (mature sequence rno-miR-378a-5p: 4-cuccugacuccagguccugugu-25) was synthesized and provided by Ruibo Biotechnology Corporation (Guangzhou, China). To obtain MSCs overexpressing miR-378 effectively, third-passage MSCs were transfected with miR-378 mimic using Lipofectamine™ 2000 (catalogue number 11668019; Invitrogen, Carlsbad, California, USA) according to the manufacturer’s instructions. Opti-MEM (catalogue number 31985070; Invitrogen) was applied as transfection medium and the medium was changed 4 hours after transfection. Total RNA and cell lysate were collected for the indicated assays.

### Hypoxia/reoxygenation treatments of MSCs

Twenty-four hours after transfection, cells of the miR-378 group (MSCs transfected with miR-378) and the control group (MSCs without transfection) were both incubated in serum-free media with 1% oxygen in a Galaxy® 48 R incubator (Eppendorf/Galaxy Corporation, Connecticut, USA) at 37°C for 24 hours and then exposed to normoxic conditions (20% oxygen) for another 24 hours. Normoxia was used as negative control during the experiments for the two groups.

### Survival and apoptosis evaluation of MSCs

After the above treatments, the MSCs of different groups were collected and suspended in complete culture medium. The growth curve and MTS assay (cellTiter96AQ, one solution cell proliferation assay, catalogue number G3582; Promega, Madison, Wisconsin, USA) were adopted to evaluate survival and proliferation ability of MSCs. A total of 1 × 10^5^ cells were equally seeded into each well on 96-well plates. After 24 hours of preconditioning, MTS was added to the medium at a final concentration of 0.5 mg/ml for 4 hours. Results were read from cellTiter96AQ at different time points (24, 48, 72 and 96 hours) respectively.

The terminal deoxynucleotidyl transferase biotin-dUPT nick end-labeling assay was applied for assessing MSC death and apoptosis *in vitro* after 24 hours of reoxygenation. Cell slices were fixed in 10% formaldehyde for 15 minutes and pretreated with 0.2% Triton X-100 for 15 minutes. The slices were then infiltrated in an equilibration buffer followed by incubating with terminal deoxynucleotidyl transferase enzyme and a nucleotide mix for another 75 minutes. All sections were examined under a florescent microscope (DMI6000B; Leica, Brunswick, Germany).

### Vascular networks formation assay of MSCs

Aliquots of human umbilical vein endothelial cells (Yiyuan Biotechnology Corporation, Guangzhou, China) were seeded onto matrigel-coated wells (catalogue number 356234; BD Corporation, New York, USA) of a 24-well plate. After hypoxia/reoxygenation treatments, MSCs were then cocultured with human umbilical vein endothelial cells in 1% fetal calf serum-supplemented Dulbecco’s modified Eagle’s medium (catalogue number SH30021.01B; Hyclone, Logan City, Utah, USA). After incubating at 37°C for 6 hours, vascular network formation was examined by a phase-contrast microscopy (CKX41, U-CTR30-2; Olympus, Tokyo, Japan), and the number of the vascular branches was quantified by randomly selecting five fields per well as described previously [28].

### Western blot analysis

Protein levels of BCL2, BAX, tumor necrosis factor alpha (TNFα), TUSC-2, vascular endothelial growth factor (VEGF) alpha, platelet-derived growth factor (PDGF) beta and transforming growth factor beta-1 (TGFβ1) were measured by western blot. After 24 hours of reoxygenation, MSCs were washed several times with phosphate-buffered saline before collection and lysing with modified RIPA buffer. Cells were completely lysed after repeated vortexing and supernatants were acquired though centrifugation at 14,000 × *g* for 20 minutes. Proteins were then resolved by SDS-PAGE and subsequently transferred to a polyvinylidenedifluoride membrane (IPVH00010; Millipore, Boston, MA, USA) before incubating with primary antibodies overnight at 4°C. The membranes were subjected to three 5-minute washes with Tris-buffered saline–Tween and then incubated with anti-IgG horseradish peroxidase-conjugated secondary antibody (Southern Biotech, Birmingham, Alabama, USA) for 60 minutes at room temperature. After extensive washing, bands were detected by enhanced chemiluminescence. The band intensities were quantified using image software (image J 2×, version 2.1.4.7).

### Quantitative real-time polymerase chain reaction

Total RNA was isolated from MSCs with hypoxia/reoxygenation treatments using a Trizol reagent (Invitrogen) followed by digestion with RNase-free DNase (Promega). The concentration and integrity of total RNA were estimated and the real-time polymerase chain reaction (RT-PCR) was conducted on an ABI PRISM® 7500 Sequence Detection System using SYBR Green qPCR SuperMix (Invitrogen). The primers are described in Table 1. Specific products were amplified and detected at 95°C for 10 minutes, followed by 40 cycles at 95°C for 15 seconds, and at 60°C for 30 seconds, at which point data were acquired. The miR-378 expression level was quantified using probes for miR-378 and U6 RNA (internal control). The relative level of miRNA was calculated using the 2^-ΔΔCt^ method. For the assays of the molecules examined, the results were quantified as the threshold cycle of each target gene and normalized into the ΔCt value. Quantifications of fold-change in gene expressions were also performed using the 2^-ΔΔCt^ method.

**Table 1:** **Primers for quantitative real-time polymerase chain reaction**

Gene name	Forward primer (5′ to 3′)	Reverse primer (5′ to 3′)
PDGFβ	GCCAGCTAGCAGGGAATACT	GGAGTTCATGTCTTCCACGAT
TNFα	TGAAGTAGTGGCCTGGATTGC	GACATTCCGGGATCCAGTGA
TUSC-2	ATGCCTGGTTCCTAGTTACTTG	AACGGCTGAAATGCTCTGA
VEGFα	AGATTCTGCAAGAGCACC	AAGGTCCTCCTGAGCTAT
TGFβ1	TGCTTCAGCTCCACAGAGAA	TGGTTGTAGAGGGCAAGGAC
BCL2	ATCCAGGATAACGGAGGCTG	CAGGTATGCACCCAGAGTGA
BAX	GGCGAATTGGAGATGAACTG	TGCCATCAGCAAACATGTCA
β-actin	AGGGAAATCGTGCGTGACAT	GAACCGCTCATTGCCGATAG

PDGF, platelet-derived growth factor; TGF, transforming growth factor; TNF, tumor necrosis factor; VEGF, vascular endothelial growth factor.

### Statistical analysis

All quantitative data are described as mean ± standard deviation. The significance of differences among groups was determined by analysis of variance and Scheffe’s multiple-comparison techniques. Comparisons between time-based measurements within each group were performed with analysis of variance for repeated measurements. *P* <0.05 was considered statistically significant.

## Results

### Mesenchymal stem cells survival was enhanced post transfection of miR-378

**Figure 1:**
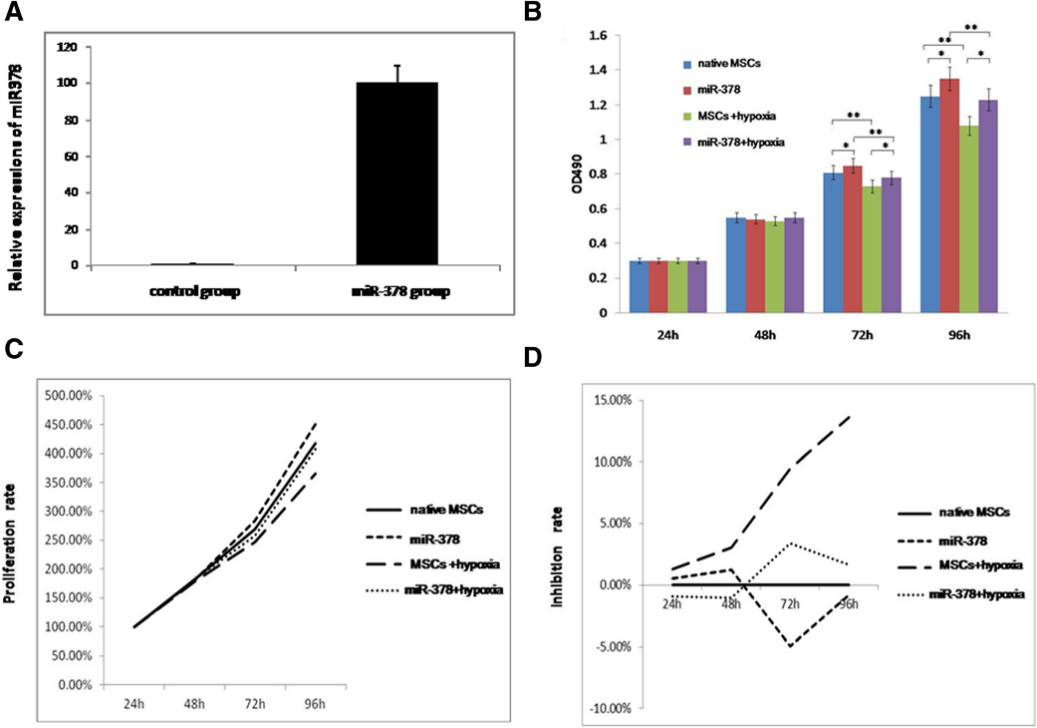
**Mesenchymal stem cell proliferation after the transfection of microRNA-378. (A)** miR-378 expression status after transfection with microRNA (miRNA) mimic detected by quantitative real-time polymerase chain reaction. **(B)** Mesenchymal stem cell (MSC) proliferation rate tested by MTS assay (cellTiter96AQ, one solution cell proliferation assay, catalogue number G3582; Promega, Madison, Wisconsin, USA). *P < 0.05; **P < 0.01. **(C)** Proliferation rate = optical density (OD) values at other time points divided by OD value at the beginning × 100% (same sample). **(D)** Inhibition rate = (1 – OD value divided by native MSC group OD value) × 100% (same time). Native MSCs, MSCs cultured in normoxic conditions; miR-378, miR-378-transfected MSCs cultured in normoxic conditions; MSCs + hypoxia, MSCs cultured in hypoxic conditions; miR-378 + hypoxia, miR-378-transfected MSCs cultured in hypoxic conditions.

miR-378 expression status after transfection with miR-378 mimic was confirmed by quantitative RT-PCR (*P* < 0.01; Figure 1A) The two experimental groups presented a lower growth and proliferation rate under a hypoxic environment at 72 hours and 96 hours, while both groups showed a higher inhibition rate in this condition (*P* < 0.01; Figure 1B, C, D). The miR-378 group displayed a more rapid growth under hypoxic environment at 72 hours and 96 hours in contrast with the control group (0.78 ± 0.02 vs. 0.73 ± 0.02, *P* < 0.05; 1.22 ± 0.03 vs. 1.08 ± 0.03, *P* < 0.05) (Figure 1B). Moreover, the transfected cells had a higher proliferation rate (257.33% vs. 246.67%, *P* < 0.01; 406.84% vs. 365.39%, *P* < 0.05) (Figure 1C). The inhibition rate of the miR-378 group was lower under a hypoxic environment at 72 hours and 96 hours (3.73% vs. 9.47%, *P* < 0.01; 1.63% vs. 13.60%, *P* < 0.01) (Figure 1D), indicating that miR-378 could alleviate the inhibition of MSC proliferation induced by hypoxia–ischemia.

### Hypoxia-induced apoptosis in MSCs was alleviated after miR-378 transfection

**Figure 2:**
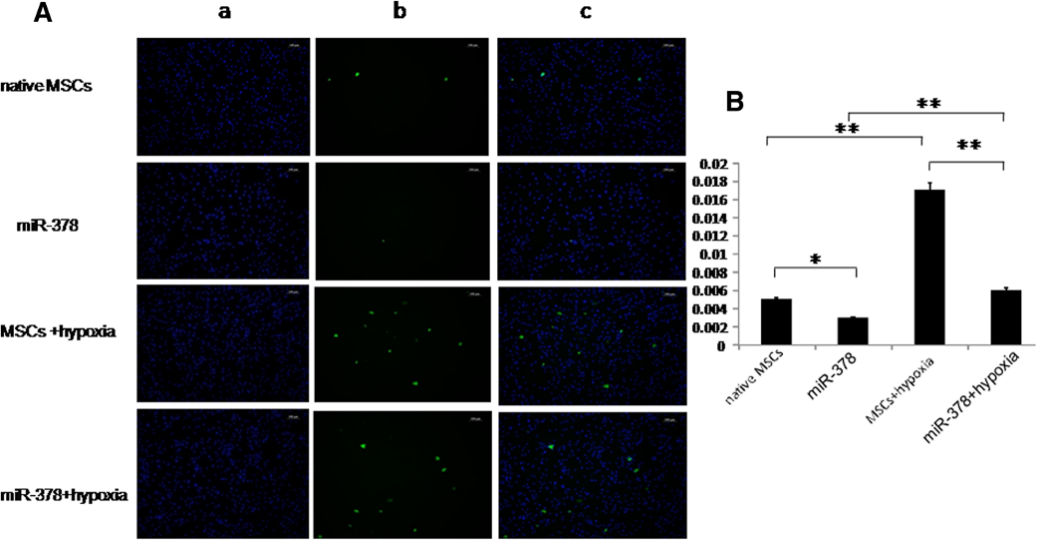
**microRNA-378 transfection reduced hypoxia-induced apoptosis in mesenchymal stem cells. (A)** Apoptosis of mesenchymal stem cells (MSCs) was detected and assessed by terminal deoxynucleotidyl transferase biotin-dUPT nick end-labeling (TUNEL) staining. lane a, DAPIr staining localization; lane b, TUNEL staining; lane c, overlap figure of lanes a and b. **(B)** Comparison of apoptotic rates between groups after miR-378 transfection in normoxic and hypoxic environments. *P < 0.05, **P < 0.01. Native MSCs, MSCs cultured in normoxic conditions; miR-378, miR-378-transfected MSCs cultured in normoxic conditions; MSCs + hypoxia, MSCs cultured in hypoxic conditions; miR-378 + hypoxia, miR-378-transfected MSCs cultured in hypoxic conditions.

The two groups both exhibited a higher apoptosis rate under the hypoxic environment (0.50 ± 0.10% vs. 1.7 ± 0.20%, *P* < 0.01; 0.30 ± 0.10% vs. 0.60 ± 0.40%, *P* < 0.01) (Figure 2B). Compared with the control group, terminal deoxynucleotidyl transferase biotin-dUPT nick end-labeling staining showed that the apoptosis rate of the miR-378 group was significantly decreased under normal (0.30 ± 0.10% vs. 0.50 ± 0.10%, *P* < 0.05; Figure 2A, B) and hypoxic (0.60 ± 0.40% vs. 1.70 ± 0.20%, *P* < 0.01; Figure 2A, B) environments.

### Angiogenesis ability of MSCs was promoted after miR-378 transfection

**Figure 3:**
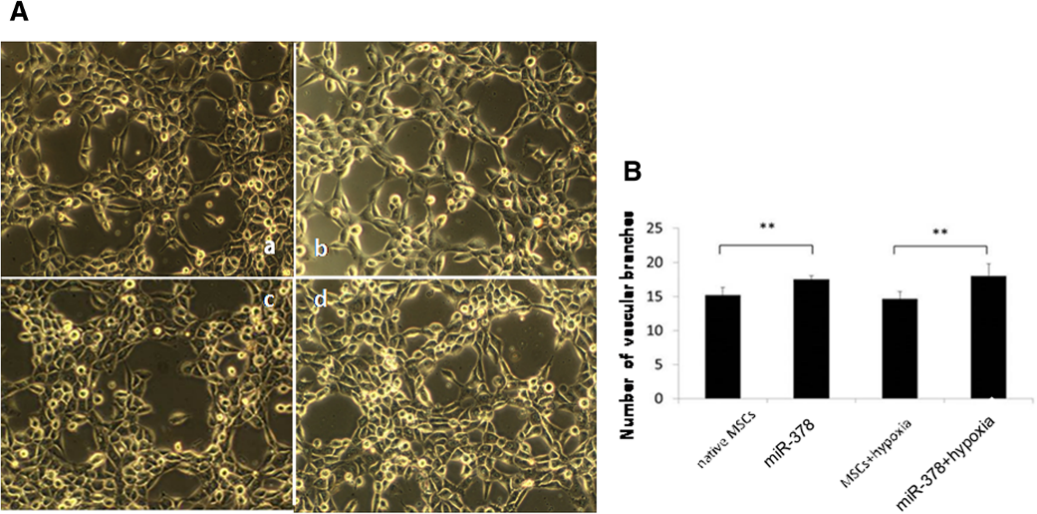
**microRNA-378 promoted the angiogenesis ability of mesenchymal stem cells.** Comparison of the numbers of vascular branches between the two experimental groups in normoxic and hypoxic conditions *in vitro*. **(A)** Vascular formation of the different groups *in vitro* (magnification × 100): (a) native MSCs, MSCs cultured in normoxia conditions; (b) miR-378, miR-378-transfected MSCs cultured in normoxia conditions; (c) MSCs + hypoxia, MSCs cultured in hypoxic conditions; (d) miR-378 + hypoxia, miR-378-transfected MSCs cultured in hypoxic conditions. **(B)** Comparison of the numbers of vascular branches between groups. **P < 0.01, transfected versus nontransfected MSCs.

Once cocultured with human umbilical vein endothelial cells, MSCs transfected with miR-378 formed a larger number of vascular branches on matrigel. Under normoxic conditions, the numbers of vascular branches in the miR-378 group were obviously higher than in the control group (17.50 ± 0.55 vs. 15.17 ± 1.17, *P* < 0.01). The same phenomenon could be observed when the two groups of cells were exposed to a hypoxic–ischemic environment (18.00 ± 1.79 vs. 14.67 ± 1.03, *P* < 0.01) (Figure 3).

### Expression of apoptosis-related genes was decreased after miR-378 transfection

**Figure 4:**
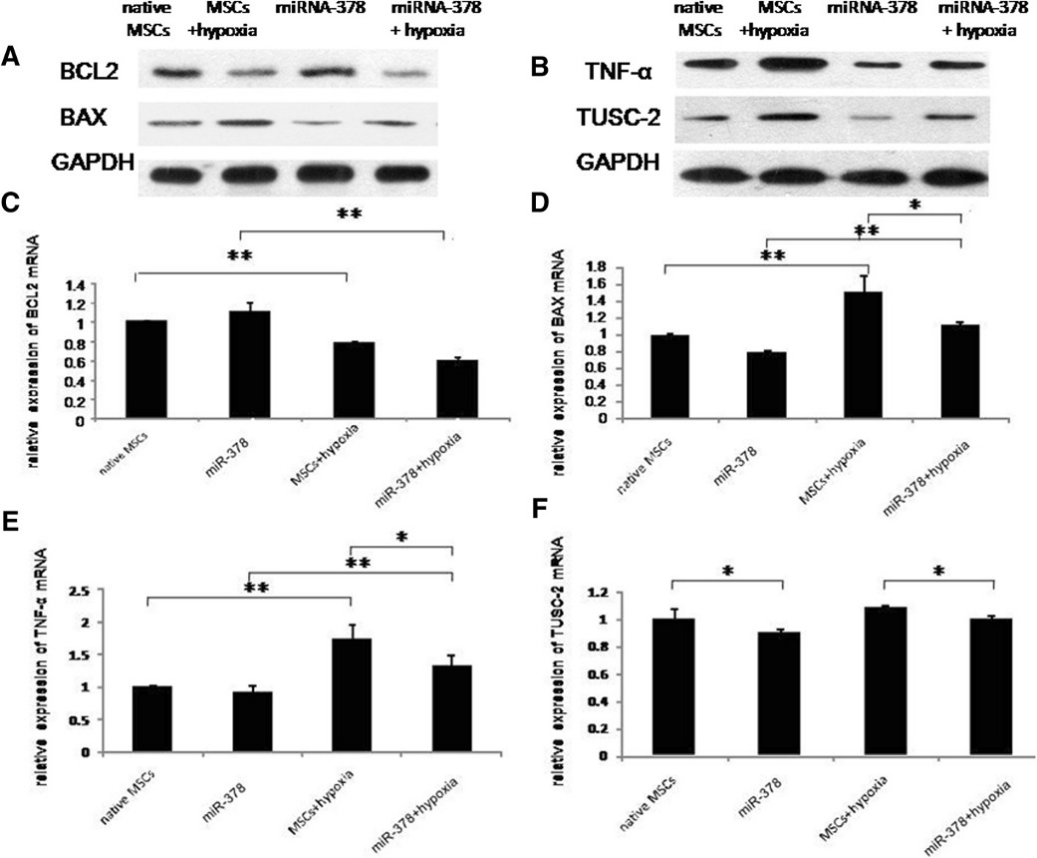
**microRNA-378 decreased the expressions of apoptosis-related genes.** Changes of apoptosis-related genes under different conditions in the two experimental groups. Western blot analysis **(A)**, **(B)** and quantitative real-time polymerase chain reaction **(C)**, **(D)**, **(E)**, (F) were performed to explore the expressions of BCL-2, BAX, tumor necrosis factor alpha (TNFα) and TUSC-2 after cells of the two experimental groups were exposed to different conditions. **P < 0.01. GAPDH, glyceraldehyde 3-phosphate dehydrogenase; miRNA, microRNA; MSC, mesenchymal stem cell.

The expression of apoptosis-related genes was examined by western blot and quantitative RT-PCR in both groups after cells experienced hypoxia/reoxygenation or were exposed to normoxic (20% oxygen) conditions (Figure 4). Detection of the BCL2 family proteins showed that the BCL2 level was decreased, accompanied with an elevated expression of BAX in the two experimental groups under a hypoxic environment (Figure 4A, C, D). There was no difference of the expression of BCL2 between the miR-378 group and the control group under normoxic and hypoxic environments (Figure 4A, C), whereas BAX expression was reduced in the miR-378 group under a hypoxic environment (Figure 4A, D). The TNFα level was increased under the hypoxic environment in the two groups (Figure 4B, E). In the miR-378 group, TNFα protein expression was decreased under the normoxic environment (Figure 4B) and its expression was downregulated both on mRNA and protein level in hypoxic conditions (Figure 4B, E). TUSC-2 expression was enhanced on the protein level under a hypoxic environment in the two groups (Figure 4B). Its level was downregulated in the miR-378 group compared with the control group under normoxic and hypoxic environments (Figure 4B, F).

### Expression of angiogenesis-related genes was enhanced after miR-378 transfection

**Figure 5:**
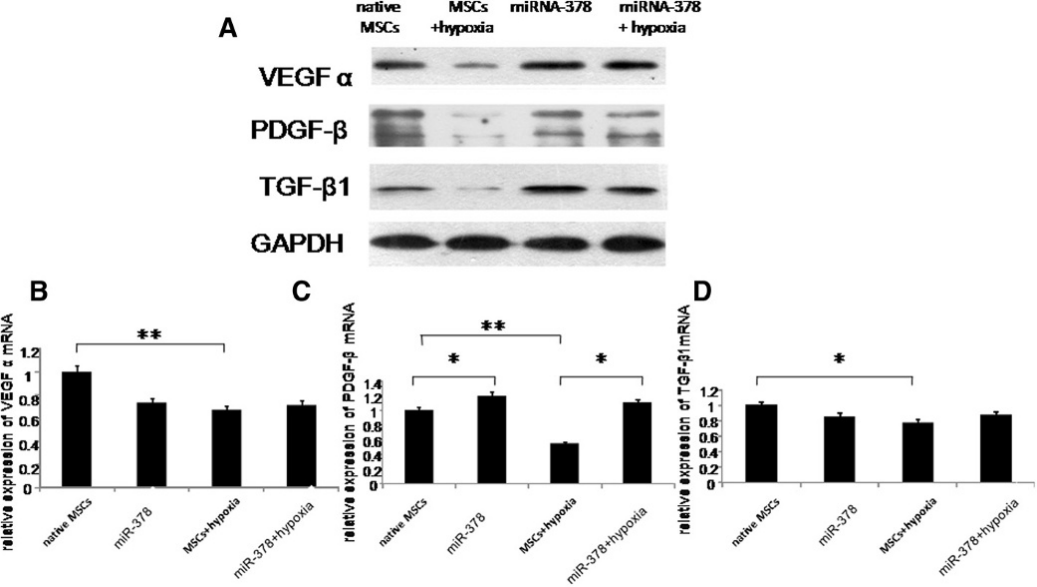
**microRNA-378 enhanced the expression of angiogenesis-related genes.** Changes of angiogenesis-related genes under different conditions in the two experimental groups. Western blot analysis **(A)** and quantitative real-time polymerase chain reaction **(B)**, **(C)**, **(D)** were performed to explore the expression of VEGFα, PDGFβ and TGFβ1 after cells of the two experimental groups were exposed to different conditions. *P < 0.05, **P < 0.01. GAPDH, glyceraldehyde 3-phosphate dehydrogenase; miRNA, microRNA; MSC, mesenchymal stem cell; PDGF, platelet-derived growth factor; TGF, transforming growth factor; VEGF, vascular endothelial growth factor.

Angiogenesis-related genes were detected the same way as described above. The expression of VEGFα, PDGFβ and TGFβ1 was decreased in the control group under hypoxic conditions (Figure 5). However, no differences in their expressions were observed in the miR-378 group when exposed to the same environment (Figure 5). Western blot analysis showed that the expression of VEGFα, PDGFβ and TGFβ1 was increased in miR-378 transfected MSCs under both normoxic and hypoxic conditions (Figure 5A). Quantitative RT-PCR indicated that there was an upregulated expression of PDGFβ on the mRNA level (Figure 5C), whereas no differences could be discovered in the expressions of VEGFα and TGFβ1 genetically (Figure 5B, D).

## Discussion

The present study demonstrates that MSCs transfected with miR-378 showed higher viability and a lower apoptosis rate under hypoxic–ischemic conditions. Moreover, their capacity for blood vessel formation was improved. The expression of proapoptosis genes was decreased whereas angiogenesis-related genes were found to be upregulated after miR-378 transfection.

MSCs are multipotent progenitor cells with the potential to differentiate into various cell types, including cells of the cardiovascular lineage [7]. MSCs can be easily isolated and expanded *in vitro*, making them an optimal therapeutic approach for cardiovascular disease [29]. In spite of this, the low survival rate of MSCs after transplantation *in vivo* restricts their therapeutic efficiencies [13, 14]. Studies have indicated that hypoxia and serum deprivation can lead to poor viability and apoptosis of MSCs via triggering a strong inflammatory and oxidative stress reaction and an overproduction of proapoptosis factors [15, 30]. Diverse methods have been applied to improve MSCs survival under hypoxic and ischemic conditions [16, 31]. miRNAs play a crucial role in cell proliferation, differentiation and apoptosis [17]. It has been reported that transfection of specific miRNAs enhances MSCs survival and their capability for lineage differentiation, making them more conducive to the repair of infarct injury [32]. Prior studies have demonstrated that miR-378 facilitates cancer cell migration and promotes their survival [21]. In this study, we investigated the effects of miR-378 on MSCs survival and proliferation. The results indicated that MSCs could be efficiently transfected with miR-378 without affecting cells viability. We found that MSCs viability was attenuated under the hypoxic condition in the two experimental groups. Cells transfected with miR-378 showed more rapid growth and a higher proliferation rate after 24 hours of hypoxia/reoxygenation. Examination of the apoptosis of MSCs showed that the number of terminal deoxynucleotidyl transferase biotin-dUPT nick end-labeling-positive cells was significantly decreased in the miR-378 group. In addition, MSCs transfected with miR-378 presented a distinctly lower apoptosis rate compared with the control group, suggesting that miR-378 might exert anti-apoptosis roles in a hypoxic–ischemic environment *in vitro*.

Evidence has revealed that miR-378 improves cell survival and inhibits apoptosis via regulation of various signaling networks and apoptosis-related genes [24]. miR-378 may reduce hydrogen peroxide and hypoxia-induced cell death by suppressing the expression of IGF-1R and its downstream AKT signaling cascade [24]. The proteins of the BCL2 family are well known as key regulators of cell death and apoptosis. Increasing evidence sustains that BCL2 family members are the targets of numerous miRNAs [33]. In the present work, we examined several BCL2 family proteins including BCL2 and BAX. We found that BCL2 was downregulated while BAX was upregulated in the two experimental groups under the condition of hypoxia exposure. In the miR-378 group, there was no alteration of the expression of BCL2 both under the normoxic and hypoxic conditions, while the BAX level was decreased in the hypoxic condition, implying that miR-378 could attenuate the apoptosis of MSCs via suppressing the proapoptosis genes of the BCL2 family. In addition to the BCL2 family, TUSC-2 (also known as Fus-1) is a potent proapoptotic gene [34]. The expression of the miR-378/miR-378 hairpin enhances cell survival through targeting the transcription factor SuFu and Fus-1 [21]. Moreover, miR-378 is capable of reducing the expression of TUSC-2 at the protein level [21]. TNFα is another pivotal molecule that initiates the process of MSC apoptosis [35]. TNFα represses the expression of proinflammatory genes by activating nuclear factor-κB [36]. TNFα can also stimulate the caspase cascade related to cell apoptosis [37]. In this study, the expression of TUSC-2 protein was upregulated and the TNFα level was enhanced in the two groups under hypoxic environments. Both the expressions of TUSC-2 and TNFα were downregulated in miR-378-transfected MSCs under a hypoxic environment, implicating that miR-378 may relieve hypoxia-induced apoptosis via downregulating TUSC-2 and TNFα.

miRNAs can exist in a lineage-specific pattern and dominate the fate of stem cell differentiation [38]. Several miRNAs have already been found to modulate cardiovascular cell differentiation [20, 39]. Previous studies have confirmed that miR-378 plays an important role in blood vessel formation [40, 41]. In the present study, we demonstrated that MSCs transfected with miR-378 formed a larger number of vascular branches on matrigel, indicating that miR-378 could enhance the capability of MSCs differentiation into vascular lineage.

miRNAs most probably control cell fate via regulating numerous genes and pathways [17, 20]. VEGF has been identified as a predominant mediator of angiogenesis [42]. VEGF hampers post-hypoxic MSC death and improves MSCs survival and regeneration in hostile environment of post-ischemic tissues [43, 44]. miR-378 is involved in the expression of VEGF [45]. miR-378 binds to the 3′ untranslated region of VEGF to compete with miR-125a for the same seed region and strengthens VEGF expression [45]. In this study, VEGFα expression was found to be declined in the control group after hypoxia exposure and this alteration could be mitigated after miR-378 transfection. In addition, VEGFα expression was increased at the protein level in the miR-378 group in hypoxic conditions. The abovementioned results supported that MSCs might strengthen VEGF expression in the context of hypoxia exposure. PDGF and TGFβ1 secreted by MSCs profoundly impact vascularization as well as proliferation of MSCs [20, 46]. We found that the expression of PDGFβ and TGFβ1 was also downregulated under hypoxic conditions in the control group; PDGF receptors can effectively induce MSCs neovascularization *in vivo*[47]. When the expression of PDGF is depressed, pathways associated with angiogenesis of MSCs are correspondingly obstructed [48]. miRNAs modulate PDGF receptor expression upon ligand stimulation through direct interaction with the 3′ untranslated region of PDGF receptor in cardiomyocytes [49]. In the present work, PDGFβ was increased in MSCs transfected with miR-378 under normoxic and hypoxic conditions, indicating that miR-378 might improve PDGF expression in this condition. MSCs have the potential to differentiate into smooth muscle cells, which makes them an ideal cell source for the construction of tissue-engineered vascular grafts [20]. TGFβ1 is a cytokine with versatile functions [50]. This cytokine can induce smooth muscle markers in MSCs, subsequently driving MSCs toward a smooth muscle cell phenotype [51]. TGFβ1 signaling has important functions in the regulation of MSCs at both transcriptional and post-transcriptional levels [52]. High concentrations of TGFβ1 contribute to the formation of nestin-positive MSC clusters, resulting in shaping of marrow osteoid islets accompanied by high levels of angiogenesis [53]. miRNAs have been proved to mediate TGFβ-induced signaling circuit loops in the pathological process of human diseases [54]. They regulate TGFβ1 expression as well as the TGFβ1-induced epithelial–mesenchymal transition phenomenon [55, 56]. In this study, we found that miR-378 transfection notably intensified the expression of TGFβ1, which could play a crucial role in promoting MSC differentiation into blood vessels. It has been recently reported that crosstalk between PDGFβ and TGFβ1 pathways is essential for mediating MSC mechanics and matrix interactions [57]. Both PDGFβ and TGFβ1 were discovered to be increased in MSCs transfected with miR-378. Therefore, we inferred that TGFβ1 in combination with PDGF might further amplify the formation of vasculature in a hypoxic–ischemic environment.

There are some limitations to this study. MSCs without transfection were applied as the negative control; experiments using scrambled miRNA as a control for the miR-378 transfection would further be performed to eliminate nonspecific effects of transfection and make the data more convincing. As this study was conducted *in vitro*, the affection of miR-378 on MSCs *in vivo* is still lacking. How miR-378 could act on MSCs survival and differentiation in the injured heart in animal models would be further explored in our later work. In addition, although several molecules that might possibly participate in promoting MSCs survival after miR-378 transfection have been investigated in this paper, the elaborate downstream signaling pathways implicated should be searched for in the future studies in order to clarify the underlying mechanisms of the biological functions of miR-378.

## Conclusions

In summary, this study showed that miR-378 could promote MSCs survival and the cells’ ability of vascularization under hypoxic–ischemic conditions *in vitro*. miR-378 might function on apoptosis-related and angiogenesis-related genes. Further explorations of the implications of miR-378 on MSCs *in vivo* will provide new mentalities for the treatment of ischemic heart disease based on MSCs transplantation.

## Abbreviations

miR-378: microRNA-378
miRNA: microRNA
MSC: mesenchymal stem cell
PDGF: platelet-derived growth factor
RT-PCR: real-time polymerase chain reaction
TGFβ1: transforming growth factor beta-1
TNFα: tumor necrosis factor alpha
VEGF: vascular endothelial growth factor.
